# Global challenges and globalization of bioethics

**DOI:** 10.3325/cmj.2013.54.83

**Published:** 2013-02

**Authors:** Farida Nezhmetdinova

**Affiliations:** Philosophy and Law Department, Kazan State Agrarian University, Kazan, Russia

## Abstract

This article analyzes problems and implications for man and nature connected with the formation of a new architecture of science, based on the convergence of nanotechnology, biotechnology, information technology, and cognitive science (NBIC). It also describes evolution and genesis of bioethics, a scientific discipline and social practice with a special role of ethical management of potential risks of scientific research. The aim was to demonstrate the necessity of bioethical social control in the development of a global bioeconomy driven by NBIC technologies.

As humankind is entering the 21st century, it is bringing along all the achievements and changes that were realized in the previous centuries, as well as its unfulfilled potentials. In the technological era, the speed of change is so high that humankind does not have enough time to comprehend and learn (1-3). This is, in my opinion, one of the main reasons for the so called civilization crisis. Motive forces of this process are the following global trends:

1. Need for energy and raw materials

2. Need to solve the problem of hunger

3. Incessant fight against diseases and protection of health of humans, animals, and plants

4. Aspiration of the population to achieve a new quality of life

5. Search for new technological platforms of innovative development in the conditions of the global competition

The global problems that we speak about arose in the 20th century. What do these problems signify today, in the 21st century?

Primarily, the concept of “global” has changed. Practically all global problems reflect on regional and local levels. Therefore the term “glocal” is emerging to represent the natural process of projection of global problems on the local level considering the geographical, cultural, political, national, and religious features of the region. In consequence, it is important to analyze possible positive and negative impacts of global problems on local communities.

Second, with the emergence of nanotechnology, biotechnology, information technology, and cognitive science (NBIC) technologies (4,5), humankind has started to change the way it approaches communications, social interactions, and its own biogenetic nature. Although we, by creation of uncontrollable new technological possibilities, assume the role of God, we unfortunately do not take up the responsibility for the consequences of such aspirations. Revolutionary breakthroughs in NBIC technologies give access not only to “nuclear,” but also to the “biogenetic” threats to humanity (3,6,7).

There is a search for a unified human platform to solve the global problems, face the consequences of the artificial/biological blend, and preserve fundamental bases of humanity and nature (2,8). As the possibility for the extension of life arises, the most existential questions of humankind come into focus, and the vital need for solutions is becoming clear.

The increased risks to human life as it is now understood put forward some imperative questions:

Why does changing of the fundamental bases of a person’s life turns his or her environment to a “globalized society of risks”?

Where can we find a uniform system for all moral coordinates and what is the role of global bioethics in its achievement?

Is the question of fundamental bases of human life really central for the world intellectual thought of the 21st century?

Are there positive scenarios for the future of human civilization?

We will briefly consider the main “disturbing” signals broadcast by the “glocal” problems.

Post-industrial consumer society of the 21st century offers vast possibilities for the use of transgenic organisms. The use of genetic bioengineering could transform global flora and fauna to a planetary network of biofactories, biofarms, and bioreactors for production of goods and services. At the same time, the question of biological safety arises, understood as preservation of biological essence of live organisms, biological qualities, backbone communications and characteristics, and prevention of a large-scale loss of biological integrity (9). Achievements of technical progress are shown today by wide application of artificial organs as replacing elements for the human body. Medical preparations are capable of correcting the characteristics of personality, alter thoughts and emotions, and even change the person’s gender (10). Many successes of genetic engineering, stem cell technology, and in vitro fertilization are ethically tolerated by the international organizations and the states. What can the consequences be?

**1.** Changing the meaning of life itself. The traditional views on the relationship between a person and its material surroundings, the clear difference between life and death, the distinction between the simulated object and the biological being, are being challenged with the coming of new technologies. The total reorganization of the human body with the incorporation of artificial elements changes our interrelations with physical space and time.

**2.** Changes in evolution. A revolution in biology, generating biomedical technologies (genetic engineering, implantology, stem cell engineering, cloning, etc) has brought into question the natural selection of individuals. The evolutionary potential put forward by this trend results in modern persons’ relinquishing some inherent biological properties, acquiring “non-human” qualities, and changing the anthropomorphous shape of civilization.

Some researchers declared the beginning of a new phase in the development of technological civilization – “risk society” or “society of globalizing risks” (1,2,11,12). What choice will humankind make? The great potential and real danger of modern biotechnological achievements makes the social and regulatory role of bioethics very important (13-20).

Why did bioethics become global?

• it is an interdisciplinary platform of dialogue

• it is a form of social regulation of risks brought about by changes in all branches of society and NBIC technologies

• it is a constructive means of communications between power, business, scientists, and civil society

• it supports and promotes scientific research and the social projects directed at preservation of health and well-being of people and nature

• it is a humanitarian expertise, “internal optics” of moral relation to life and to categorical imperative of bio economy

Proceeding from my definition of “bioethics as pursuit, assessment, and a choice of criterion of the moral relation to live*”* (9,21) it is possible to define three levels:

1. Theoretical level – the interdisciplinary and complex analysis of ethical and axiological aspects of different life activities. There are numerous concepts and theories of moral relation of a person to life (eg, humanitarian, utilitarian, deontological, etc). There are also historical, cultural, and social implications that need to be taken into account when assessing these problems. The features of recoverability and irrevocability of moral decision-making as an axial principle depend on the available technological possibilities of transformation of life systems.

2. The bioethical aspects can be applied to activity types such as medicine, science, politics, sports, agriculture, etc. These systems are operated and regulated by professional codes and moral principles, laws, and regulations through a prism of a public discourse (22). On this level, it is possible to speak about different types of bioethics which we observe today: biomedical ethics, agro bioethics, cyber bioethics, sports bioethics, ecological bioethics, global bioethics, scientific bioethics, etc (21).

3. Practical or clinical bioethics – concrete bioethical examination and assessment of the problem, demanding a moral choice here and now, in a situation (as a rule) not previously experienced. This application is summarized in the term bioethical know-how. Examples of such decisions create a bank of bioethical casuistry (23,24), which becomes a practical and methodological basis of “advancing knowledge,” providing comprehension and reformative impact on “small standard and valuable systems.”

## Conclusion

1. The field of bioethics grew out of the need for interpreting and controlling global changes at a time of significant transformation. The achievements of modern science and the result of globalization influenced the speed of bioethical development. It also mediated the collaboration of the international community with the goal of finding the solution to global problems.

2. Bioethics represents a new type of scientific knowledge, which relies on procedures and methods of “advancing experience.” At the same time, there is theoretical analysis and accumulation of new knowledge, stimulation of public discussion, and practical adoption of moral decisions.

3. Today the global role of bioethics needs to develop as the formation of: 1. Ideals, norms, principles; 2. Humanitarian expertise; 3. Scientific discipline; 4.Educational topics; 5. Ethical committees; 6. Experts on bioethics.

## References

[1] Fukuyama F. Our post-human future. Consequences biotechnological revolution. Moscow: AST Publishing House; “Lux” Co, 2004.

[2] Nesbit J. High technology, deep humanity. Technologies and our searches of sense. Moscow: Nuclear heating plant: Transitbook Press; 2005.

[3] Shaykhudinov RG (2007). Modern technologies of the power.. Philosophy Questions..

[4] Kovalchuk MVConvergence of sciences and technologies – break in future. The Russian nanotechnologies20016;13-24

[5] 2045: The year man becomes immortal. Available from: http://www.time.com/time/health/article/0,8599,2048138,00.html*.* Accessed: January 23, 2013.

[6] Tishchenko PD. Bio-power during an era of biotechnologies. Moscow: IFRAN Press; 2001.

[7] Vodopyanova E.Other science: order innovative society. Free thought20074:135

[8] Philosophy of biomedical research: ethos of sciences of the beginning of the third millennium. Moscow: Institute of the person of the Russian Academy of Sciences; 2004.

[9] Nezhmetdinova FT. An urgency of humanitarian examination in a role of «public reason» and as factor of preservation human potential. /Prospects of modern development: Materials of the Russian scientific conference (on December 10-11, 2003). Section of interdisciplinary research studies “Public and private spaces of modern society”.–Part 5. – Kazan (Russia): Publishing House of the Kazan State Technical University; 2004.

[10] Beauchamp TL. Principle of biomedical ethics. Oxford (UK): Oxford University Press; 1994.

[11] Beck U. Risk society. On a way to another modern. Moscow: AST Press; 2000.

[12] Jonas G. Responsibility principle. Ethics experience for a technological civilization. Science as personal experience. Oak Ridge (TN): Iris press; 2004.

[13] Potter VR. Bioethics: Bridge to the future. Englewood Cliffs (NJ): Prentice-Hall; 1971.

[14] Reich WT, editor. Encyclopedia of bioethics, volume 1. New York (NY): New York Press; 1995.

[15] Potter VR. Global bioethics. East Lansing (MI): Michigan State University Press; 1988.

[16] Hellegers A (1971). Bioethics center formed.. Chem Eng News.

[17] Reich WT (1994). The word «Bioethics»: Its birth and the legacies of those who shaped it.. Kennedy Inst Ethics J.

[18] Callahan D. Bioethics. In: Reich WT, editor. Encyclopedia of bioethics, volume 1. New York (NY): New York Press; 1995.p. 247-8.

[19] Charlsworth M. Bioethics in liberal society. Cambridge (UK): Cambridge University Press; 1993.

[20] Engelhard HT Jr. The foundation of bioethics. Oxford (UK): Oxford University Press; 1994.

[21] Nezhmetdinova FT, Islanova NN. Law and medicine: bioethical bases. – Kazan Russia): Press House; 1998.

[22] Bakshtanovsky VI Somogonov YVApplied ethics: idea, bases, and way of existence. Philosophy questions20079:39-40

[23] Shannon TA. An introduction to bioethics. Mahwah (NJ): Paulist Press; 1997.

[24] Hare RM. Essays on bioethics. Oxford (UK): Clarendon Press;1993.

